# Current Updates on Surgical Management of Patients with Early-Stage Cervical Cancer

**DOI:** 10.3390/cancers17132259

**Published:** 2025-07-07

**Authors:** María Clara Santía, Tommaso Meschini, Heng-Cheng Hsu, Paula Mateo-Kubach, Elise M. Yates, Karolina Kilowski, Behrouz Zand, Rene Pareja, Pedro T. Ramirez

**Affiliations:** 1 Department of Obstetrics and Gynecology, Houston Methodist Hospital, Houston, TX 77030, USA; tommaso.meschini93@gmail.com (T.M.); ahsu@houstonmethodist.org (H.-C.H.); pmateogutierrez@houstonmethodist.org (P.M.-K.); ekyates@houstonmethodist.org (E.M.Y.); kkilowski@houstonmethodist.org (K.K.); bzand@houstonmethodist.org (B.Z.); pramirez3@houstonmethodist.org (P.T.R.); 2 Department of Obstetrics and Gynecology, Women’s and Children’s Del Ponte Hospital, University of Insubria, 21100 Varese, Italy; 3 National Taiwan University Hospital, Taipei 100, Taiwan; 4 Gynecology, Gynecologic Oncology, Clinica Astorga, Medellín, and Instituto Nacional de Cancerología, Bogotá 111511, Colombia; ajerapener@gmail.com

**Keywords:** cervical cancer, early-stage disease, radical hysterectomy, minimally invasive surgery, fertility-sparing surgery, sentinel lymph node, surgical de-escalation

## Abstract

The recommended treatment for early-stage cervical cancer (FIGO 2018 stages IA–IB2 and selected IIA1) is surgery, followed by either observation or adjuvant therapy, based on individual risk factors. Surgical management has evolved from extensive radical procedures to more conservative strategies, allowing for fertility-preserving options in appropriately selected patients. In 2018, a landmark study (LACC trial) evaluated the surgical approach to radical hysterectomy, comparing open vs. minimally invasive surgery. The results demonstrated that minimally invasive surgery was associated with worse disease-free and overall survival, leading to guidelines changes that recommend the open radical hysterectomy as the new standard of care. More recently, results from the prospective randomized SHAPE trial demonstrated that in well-selected patients with low-risk early-stage cervical cancer, recurrence rates are comparable between simple hysterectomy and radical hysterectomy. An ongoing study, the CONTESSA trial, is evaluating the role of neoadjuvant chemotherapy in the setting of fertility preservation for lesions measuring 2–4 cm. In addition, ongoing studies are evaluating different surgical approaches for both simple hysterectomy (LASH trial) and radical hysterectomy (ROCC/GOG-3043 and RACC trials), with a focus on comparing oncologic outcomes. Attention has also turned to refining lymph node assessment. Sentinel lymph node biopsy has become a standard staging strategy with reduced morbidity. The SENTICOL I-II and SENTIX/ENGOT-Cx2 trials support its safety and diagnostic accuracy in early-stage disease. This article offers a comprehensive overview of recently published prospective trials that have shaped clinical practice in the management of early-stage cervical cancer. It focuses on surgical approaches and radicality, the role of sentinel lymph node mapping, and fertility-sparing treatments. The review further draws attention to ongoing investigations and novel studies that may influence future directions in the field.

## Simple Summary

This review offers a comprehensive update on the evolving surgical approaches to early-stage cervical cancer. It summarizes recent prospective trials that have shaped current clinical guidelines, highlighting a paradigm shift from radical hysterectomy toward more conservative and fertility-preserving approaches in selected patients. The role of sentinel lymph node mapping in decreasing surgical morbidity while maintaining oncologic safety is also explored. The review supports individualized surgical decision-making based on risk stratification and emerging high-level evidence.

## 1. Introduction

Cervical cancer remains a significant global health challenge, being the fourth most common malignancy among women worldwide [1]. In the United States alone, the American Cancer Society estimates that by 2025, about 13,360 new cases of invasive cervical cancer will be diagnosed, and approximately 4,320 women are expected to die from the disease [2]. The standard approach for managing early-stage cervical cancer (FIGO 2018 stage IA1 through selected IIA1) involves primary surgical treatment. Traditionally, this has been considered a radical hysterectomy with pelvic lymph node assessment. This approach results in high cure rates and is typically followed by observation or adjuvant therapy (radiation with or without chemotherapy) if adverse pathological risk factors are present.

The surgical approach to early-stage cervical cancer has become the subject of increasing discussion in recent years. There is growing debate among gynecologic oncologists regarding the necessity of extensive surgery, including radical hysterectomy and pelvic lymphadenectomy, and the impact of surgical technique on oncologic outcomes. For instance, the advent of minimally invasive surgery (MIS)—including laparoscopy and robot-assisted techniques—has been widely embraced in gynecologic oncology for its potential to reduce perioperative morbidity. However, the unexpected findings of the Laparoscopic Approach to Cervical Cancer (LACC) trial [3] have challenged the oncologic safety of MIS for radical hysterectomy. Concurrently, there is a movement toward surgical “de-escalation” for low-risk patients. Prospective studies suggest that less radical surgeries (simple hysterectomy or conization/trachelectomy) may achieve the same oncological outcomes as traditional radical hysterectomy in carefully selected early-stage cases. Similarly, advancements in lymph node staging—particularly sentinel lymph node (SLN) mapping—aim to reduce lymphatic morbidity while preserving oncologic outcomes.

Given these developments, the surgical management of early-stage cervical cancer is in evolution. This review examines the most recent evidence from key clinical trials that guide clinical decision-making and inform updates to current treatment guidelines. It focuses on surgical approaches and radicality, the role of sentinel lymph node mapping, and fertility-sparing treatments (Table 1). Furthermore, it highlights ongoing research and emerging studies with the potential to further advance the field.

**Table 1:** Overview of key prospective surgical studies impacting cervical cancer care.

Name (Year of Publication)	Design	Aim	Stage	Number of Patients and Interventions	Results	Conclusion
*Surgical Approach*						
**LACC** **(2018)** [3]	Phase 3 multicenter randomized non-inferiority	Compare DFS between MIS and open radical hysterectomy	Early-stage (FIGO 2009 IA1 with LVSI to IB1) squamous cell carcinoma, adenocarcinoma or adenosquamous	319 to MIS (laparoscopic or robotic) 312 to open surgery	At 4.5 years DFS rate was 86.0% for patients undergoing MIS, compared to 96.5% for those who had open surgery (95% CI, −16.4 to −4.7). MIS was linked to a reduced DFS rate, with a 3-year DFS of 91.2% versus 97.1% for open surgery (HR 3.74; 95% CI, 1.63–8.58). MIS was also associated with a lower rate of OS at 4.5 years (90.6% vs. 96.2%; HR 2.71; 95% CI, 1.32–5.59).	MIS was associated with inferior DFS and OS.
*Conservative Treatment/Fertility Sparing*						
**ConCerv** **(2021)** [4]	Multicenter, prospective, single arm	Evaluate feasibility of cone or simple hysterectomy and lymph node staging	Early-stage (FIGO 2009 IA2-IB1) and low-risk: squamous cell carcinoma (any grade), or adenocarcinoma (grade 1 or 2), tumor size <2 cm, no LVSI, depth of stromal invasion <10 mm, negative imaging for metastatic disease, negative conization margins	42 conization 36 conization followed by hysterectomy 16 inadvertent simple hysterectomy	Lymph node metastases were detected in 5% of patients. The rate of residual disease in the hysterectomy specimen following conization was 2.5%. The 2-year recurrence rate was 3.5% overall (95% CI 0.9–9.0%): 2.4% conization alone, 0% conization-hysterectomy, and 12.5% unintentional hysterectomy.	Conservative surgery was safe and feasible in patients with early-stage and low-risk.
**LESSER** **(2022)** [5]	Phase 2, multicenter, randomized non-inferiority	Compare DFS between simple hysterectomy (Type A) and modified radical hysterectomy (Type B)	Early-stage (FIGO 2009 IA2-IB1), tumor size ≤2, adenocarcinoma, squamous, or adenosquamous (any grade)	20 to Simple hysterectomy 20 to Modified radical hysterectomy (92.5% (37/40) performed by open approach)	The 3-year DFS was 95% (95% CI, 68–99%) for simple and 100% (95% CI, 100–100%) for modified radical hysterectomy. OS was 90% (95% CI, 64–97%) vs. 91% (95% CI, 50–98%)	Simple hysterectomy is safe and potentially non-inferior to modified radical surgery in DFS and OS
**SHAPE** **(2023)** [6]	Phase 3, multicenter, randomized, non-inferiority	Compare cancer recurrence between radical and simple hysterectomy	Early-stage (FIGO 2009 stage 1A2-1B1) and low-risk: squamous cell carcinoma, adenocarcinoma, or adenosquamous lesion ≤2 cm, and <10 mm stromal invasion on LEEP/cone biopsy or <50% invasion on preoperative MRI.	350 to radical hysterectomy 350 to simple hysterectomy	At 3 years, pelvic recurrence occurred in 2.17% of patients who underwent radical hysterectomy and 2.52% of those who had simple hysterectomy (absolute difference 0.35%; 90% CI −1.62 −2.32).	No difference in pelvic recurrence-free survival, extrapelvic recurrence-free survival, recurrence-free survival, or OS for simple hysterectomy.
**GOG 0278** **(2025)** [7]	Phase 1/2, multicenter, prospective	Evaluate physical function and QOL before and after non-radical surgical treatment (simple hysterectomy or conization)	Early-stage (FIGO 2009 stage IA1 with LVSI and IA2–IB1), tumor size ≤2 cm; squamous cell carcinoma, adenocarcinoma, or adenosquamous	72 conization 152 simple hysterectomy	Both groups experienced temporary declines in bladder, bowel, and sexual function postoperatively, with gradual recovery. QOL improved and cancer-related worry decreased over time. Lymphedema, was reported by 12 patients (6 in each group)	Non-radical surgery is associated with excellent QOL and only small, transient declines in bladder, bowel, and sexual function.
*Lymph Nodes Status*						
**SENTICOL I** **(2011)** [8]	Multicenter, prospective, longitudinal study	Assessed the sensitivity and NPV of SLN biopsy detected by combined-labeling (technetium 99 lymphoscintigraphy and Patent Blue injection)	Early-stage (FIGO 2009 stage IA1 with LVSI and IA2–IB1), squamous cell carcinoma, adenocarcinoma, or adenosquamous	139 patients combined-labeling	SLNs successfully identified in 136 cases (97.8%; 95% CI, 93.8–99.6%). Among 25 patients with nodal metastases, 23 were true positives and 2 were false negatives, yielding a sensitivity of 92.0% (95% CI, 74.0–99.0%) and an NPV of 98.2% (111/113; 95% CI, 74.0–99.0%). No false negatives occurred in the 104 patients (76.5%) with bilateral SLN detection.	Combined-tracer SLN mapping is a feasible and clinically reliable strategy for nodal assessment.
**SENTICOL II** **(2021)** [9]	Multicenter, prospective, randomized	Compare morbidity related to SLN resection alone to SLN resection and PLND	FIGO 2009 stage IAI with LVSI, IA2, IB1, IIA1; all histologic types except neuroendocrine carcinoma	105 SLN biopsy alone 101 SLN biopsy and PLND (87.4% had FIGO stage IB1)	Lymphatic complications were lower in the SLN group (31.4% vs. 51.5%), as were postoperative neurological symptoms (7.8% vs. 20.6%). Rates of significant lymphoedema were similar.	SLN biopsy was associated with a significantly reduced rate of lymphatic morbidity compared to systematic lymphadenectomy.
**SENTIX/ENGOT-Cx2** **(2024)** [10]	Multicenter, prospective, single-arm	Assessed the oncological safety (recurrence rate) of SLN biopsy without further systematic lymphadenectomy	FIGO 2009 IA1 with LVSI to IB1 cervical cancer; tumor size <4 cm or <2 cm if fertility sparing management; all histologic types. Successful bilateral SLN detection and negativity on frozen section pathological	647 underwent SLN ultrastaging	The 2-year recurrence rate was 6%. Sentinel lymph node biopsy achieved a 92.3% bilateral detection rate, identifying nodal involvement in over 56% of cases intraoperatively and over 90% after ultrastaging.	SLN biopsy alone demonstrated confirmed non-inferiority in recurrence rates compared to standard pelvic lymphadenectomy.

DFS, disease-free survival; MIS, minimally invasive surgery; OS: overall survival; LEEP, loop electrosurgical excision procedure; MRI, magnetic resonance imaging; QOL, quality of life; NPV, negative predictive value; PLND, pelvic lymph node dissection.

## 2. Surgical Approach

### 2.1. Minimally Invasive vs. Open Surgery

Worldwide, approximately 40–50% of cervical cancers are diagnosed at an early stage [11]. Radical hysterectomy, which entails removing the uterus, cervix, upper vaginal segment, and parametrial tissue, in combination with pelvic lymph node assessment is the standard approach for patients diagnosed with early-stage (FIGO 2018 stages IA-IB2 and select IIA1) cervical cancer who have completed childbearing [12,13].

Before 2018, MIS was considered an acceptable approach for performing radical hysterectomy. At that time, multiple single-institution series and observational cohort studies suggested that MIS did not compromise oncological outcomes and was associated with improved perioperative outcomes [14,15,16,17,18,19]. In November 2018, a landmark study—the LACC trial [3], a phase 3 multicenter randomized noninferiority study—challenged what had been accepted for more than two decades as the standard treatment for early-stage cervical cancer. The study found that in patients with early-stage cervical cancer (FIGO 2009 clinical-stage IA1 disease with lymphovascular space invasion, IA2 disease, or IB1), MIS resulted in inferior disease-free survival outcomes when compared to the open surgical approach (3-year rate, 91.2% vs. 97.1%; HR for disease recurrence or death from cervical cancer, 3.74; 95% CI, 1.63 to 8.58). Furthermore, patients undergoing MIS experienced higher rates of locoregional recurrence (3-year rate, 94.3% vs. 98.3%; HR for locoregional recurrence, 4.26; 95% CI, 1.44 to 12.60). The recently published final overall survival analysis of the LACC trial demonstrated that the overall survival rate at 4.5 years was 90.6% in the MIS group compared to 96.2% in the open surgery group (HR for death from any cause: 2.71; 95% CI, 1.32–5.59; *p* = 0.007) [20] (Table 1).

As a secondary aim, the LACC trial compares intraoperative and postoperative adverse events (Common Terminology Criteria for Adverse Events, CTCAE grade ≥2) in both groups. They found that although the overall rate of intraoperative and postoperative adverse events was comparable between the two surgical approaches, certain complications differed. The open surgery group experienced higher rates of blood loss, wound-related issues, and cardiac complications. In contrast, the MIS group showed a greater incidence of vaginal vault-related events [21]. Quality of life (QOL) was also assessed as a secondary endpoint. Despite expectations that MIS would offer superior postoperative QOL compared to open surgery, outcomes were similar between the two groups at six weeks and during subsequent follow-up. Notably, a subgroup analysis revealed that the presence of moderate to severe adverse events (CTCAE grade ≥2) was associated with poorer QOL outcomes, regardless of the surgical approach [22].

Concurrent with the release of the LACC trial, a population-level study using data from the National Cancer Database (NCDB) and the Surveillance, Epidemiology, and End Results (SEER) 18-registry database, which included 2,461 patients, also reported worse oncologic outcomes for patients undergoing MIS compared to open surgery for early-stage cervical cancer. With a median follow-up of 45 months, the 4-year mortality rate was 9.1% in the MIS group versus 5.3% in the laparotomy group (HR 1.65; 95% CI, 1.22–2.22; *p* = 0.002, log-rank test). Furthermore, the increasing use of MIS after 2006 was associated with a yearly decline in 4-year relative survival of 0.8% (95% CI, 0.3–1.4; *p* = 0.01 for trend change) [23]. Prior to the LACC trial, the use of MIS increased significantly, rising from 45.6% in 2012 to 75.3% in 2017 (*p* < 0.001). However, following the publication of the study, the use of MIS dropped from 50.4% in 2018 to 11.4% in 2020 (*p* < 0.001) [24].

While the LACC trial was not designed to determine the mechanisms behind worse outcomes with MIS, emerging hypotheses suggest that variations in surgical technique could be contributory. Two ongoing prospective trials, ROCC/GOG-3043 [25] and RACC [26], are assessing the oncologic safety of robotic-assisted versus open radical hysterectomy in early-stage cervical cancer. Based on the hypothesis that tumor dissemination may result from the use of uterine manipulators or by exposure of the tumor to the peritoneal cavity during colpotomy, both studies prohibit the use of intrauterine manipulators, require tumor containment techniques, and mandate that participating surgeons have performed at least 12 to 20 robotic radical hysterectomies prior to the study.

### 2.2. Moving Toward Conservative Treatment

Radical hysterectomy carries a risk of intraoperative and postoperative complications, including hemorrhage, injury to the bladder or ureters, and the development of fistulas, all of which contribute to significant morbidity. Due to the extent and radicality of the procedure, along with the removal of the parametrium—which contains autonomic nerve fibers involved in bladder, bowel, and sexual function—cancer survivors may experience side effects that lead to functional impairments [27]. Several retrospective studies have indicated that less than 1% of patients with early-stage cervical cancer and favorable pathological features—such as tumors smaller than 2 cm, invasion depth under 10 mm, and absence of pelvic nodal metastases—exhibit parametrial involvement [28,29,30,31,32]. Building on this evidence, the ConCerv trial [4] was designed to evaluate the feasibility and oncologic safety of conservative surgery (conization or simple hysterectomy) in patients with early-stage low-risk cervical cancer (FIGO 2009 stage IA2-IB1 squamous cell carcinoma (any grade) or adenocarcinoma (grades 1–2), including tumor size under 2 cm, absence of lymphovascular space invasion (LVSI), depth of stromal invasion less than 10 mm, no radiologic evidence of metastatic spread, and clear margins following conization). Patients eligible and interested in preserving fertility were treated with conization combined with pelvic lymph node evaluation, whereas those not pursuing fertility preservation underwent simple hysterectomy along with pelvic nodal assessment. An expert gynecologic pathologist at MD Anderson Cancer Center centrally reviewed all pathologist specimens. After a median follow-up period of 36.3 months, the overall recurrence rate at two years was 3.5%. Specifically, recurrence occurred in 2.4% (1 of 42) of patients who underwent conization alone, in none (0 of 36) of those treated with conization followed by hysterectomy, and in 12.5% (2 of 16) of individuals who unintentionally received a simple hysterectomy (Table 1). Additionally, the incidence of lymph node positivity was 5%. Based on these findings—and building on the concept that conservative management of early-stage cervical cancer is a feasible and oncologically safe alternative to radical surgery—the LESSER study [5] was developed to evaluate the efficacy and safety of simple hysterectomy in patients with early-stage cervical cancer and tumors ≤2 cm. Importantly, high-risk pathological features—including lymphovascular space invasion (LVSI), grade 3 histology, and deep stromal invasion identified post-conization—were not exclusion criteria. The study included 40 participants, equally divided between those undergoing simple hysterectomy (n = 20) and modified radical hysterectomy (n = 20). At a median follow-up of 52.1 months, the 3-year disease-free survival rate was 95% (95% CI, 68–99%) for the simple hysterectomy group and 100% (95% CI, 100–100%) for the modified radical hysterectomy group (log-rank *p* = 0.30). Five-year overall survival was also comparable: 90% (95% CI, 64–97%) versus 91% (95% CI, 50–98%), respectively (log-rank *p* = 0.46). These findings suggest that simple hysterectomy is safe and potentially non-inferior to modified radical hysterectomy, providing preliminary evidence to support simple hysterectomy as a viable alternative to radical surgery in cervical cancers ≤2 cm (Table 1).

In this context, the SHAPE trial [6], published in 2024, emerged as a landmark phase III, multicenter, non-inferiority randomized controlled trial that compared radical hysterectomy and simple hysterectomy with lymph node assessment in 700 patients with low-risk cervical cancer. This study redefined the new standard of care by demonstrating that in patients with low-risk cervical cancer (FIGO 2009 stage IA2–IB1 squamous cell carcinoma, adenocarcinoma, or adenosquamous carcinoma—regardless of histologic grade or LVSI status—with tumors ≤2 cm and limited stromal invasion defined as <10 mm on LEEP/cone biopsy or <50% on preoperative MRI) simple hysterectomy is not inferior to radical hysterectomy. With a median follow-up of 4.5 years, the 3-year pelvic recurrence rates were 2.2% in the radical hysterectomy group and 2.5% in the simple hysterectomy group (absolute difference, 0.35 percentage points; 90% CI, −1.62 to 2.32), confirming non-inferiority. No statistically significant differences were observed between the two surgical groups in terms of pelvic or extrapelvic recurrence-free survival, overall recurrence-free survival, or overall survival. Notably, patients who underwent radical hysterectomy experienced higher rates of urinary complications, including incontinence (*p* = 0.003) and urinary retention (*p* = 0.0001). Patients in the simple hysterectomy group reported better quality of life and improved sexual health outcomes, with enhanced vaginal function and increased sexual activity sustained for up to two years postoperatively. Additionally, they experienced lower rates of long-term sexual dysfunction and vaginal impairment [33]. These findings provide robust evidence in favor of surgical de-escalation in low-risk cervical cancer, highlighting the potential of less radical procedures to preserve functional outcomes without compromising oncologic safety (Table 1).

Recently, the results of the GOG 278 [7] were published. This multi-institutional, international study aimed to evaluate physical function and QOL before and after simple hysterectomy or cone biopsy with pelvic lymphadenectomy for early-stage cervical cancer (FIGO 2009 Stage IA1 with LVSI and IA2-IB1 ≤2 cm). A total of 224 patients were enrolled in the study and stratified based on their fertility wishes to either conization (fertility preservation) or simple hysterectomy (fertility preservation not desired). All patients had previously undergone a cone biopsy or LEEP with a required depth of stromal invasion ≤10 mm. The study found that non-radical surgery for early-stage cervical cancer is associated with excellent QOL and only small, transient declines in bladder, bowel, and sexual function, which generally returned to baseline or improved postoperatively. Lymphedema was infrequent but observed in both groups, affecting nine patients in the simple hysterectomy group and eight in the cone biopsy group. As a secondary objective, the trial also evaluated oncologic safety, perioperative morbidity, and reproductive outcomes following conservative surgery. After a median follow-up of 37 months, the recurrence-free survival rate was 94.8% (95% CI, 89–100%), with recurrence occurring in three patients (4%) who had undergone cone biopsy, and no recurrences were reported in the simple hysterectomy group. Adverse events of grade ≥3 within 30 days of surgery were reported in one patient from the cone biopsy group and seven patients from the simple hysterectomy group. Among the 31 patients in the cone biopsy group who desired pregnancy during the study, 16 pregnancies were achieved [34]. Overall, these findings support the safety and low morbidity of non-radical surgery in early-stage cervical cancer, particularly in patients treated with cone biopsy and pelvic lymph node dissection who may still wish to preserve fertility (Table 1).

**Figure 1:**
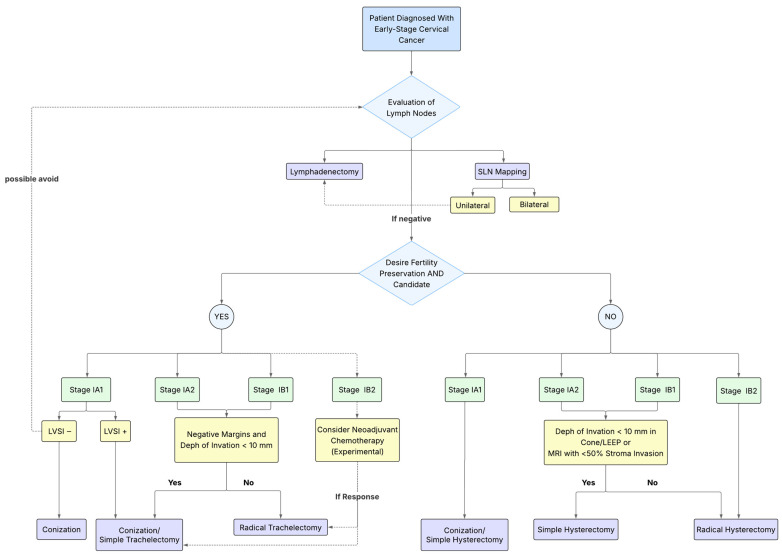
Primary treatment algorithm for early-stage cervical cancer. All staging is based on the updated FIGO 2018 staging. SLN, sentinel lymph node; LVSI, lymphovascular space invasion; LEEP, loop electrosurgical excision procedure; MRI, magnetic resonance imaging.

It is important to note that the majority of simple hysterectomies performed in ConCerv [4], SHAPE [6] and GOG 0278 [7] trials were conducted using a MIS approach. This is not surprising, given all three studies were initiated well before the LACC trial [3] raised safety concerns in 2018. Although none of these studies were specifically designed to compare the safety of MIS to laparotomy, the low recurrence rates observed are reassuring. Despite differences in patient risk profiles—particularly regarding stage and tumor size—and the relatively shorter follow-up periods, the relapse rates reported appear lower than the 7.7% recurrence rate at 4.5 years for tumors <2 cm observed in the LACC trial. Further clarification on this issue is expected from the results of the ongoing LASH trial [35], a prospective single-arm study designed to evaluate the safety and feasibility of MIS simple hysterectomy in patients with low-risk cervical cancer—defined by SHAPE criteria—who have undergone conization with clear margins. With a planned enrollment of 974 participants, the study hypothesizes that simple hysterectomy via MIS represents an oncologically safe approach in selected patients with low-risk cervical cancer (Table 2) (Figure 1).

**Table 2:** Stage-based surgical management in early cervical cancer: fertility-sparing vs. standard approaches.

FIGO 2018 Stage (Size)	Fertility Sparing		Non-Fertility Sparing	
	LVSI −	LVSI +	LVSI −	LVSI +
**Stage IA1** **(≤3 mm)**	Cone Biopsy */ Simple Trachelectomy ^&^	Cone Biopsy */ Simple Trachelectomy ^&^ + SLN/Lymphadenectomy	Cone Biopsy/ Simple Hysterectomy (Type A) ^	Simple Hysterectomy(Type A) ^/ Modified Radical Hysterectomy (Type B) ^&^ + SLN/Lymphadenectomy
**Stage IA2** **(>3–≤5 mm)**	Cone Biopsy */ Simple Trachelectomy ^&^ + SLN/Lymphadenectomy	Cone Biopsy */ Simple Trachelectomy ^&^ + SLN/Lymphadenectomy	Simple Hysterectomy (Type A) ^ + SLN/Lymphadenectomy	Simple Hysterectomy (Type A) ^ + SLN/Lymphadenectomy
**Stage IB1** **(>5–≤2 cm)**	Cone Biopsy */Simple Trachelectomy ^&^/Radical Trachelectomy ^#^ + SLN/Lymphadenectomy	Cone Biopsy */Simple Trachelectomy ^&^/Radical Trachelectomy ^#^ + SLN/Lymphadenectomy	Simple Hysterectomy (Type A) ^/Radical Hysterectomy (Type C) ^#^ + SLN/Lymphadenectomy	Simple Hysterectomy (Type A) ^/ Radical Hysterectomy (Type C) ^#^ + SLN/Lymphadenectomy
**Stage IB2** **(>2 – ≤4 cm)**	Neoadjuvant CT + Cone Biopsy/Simple Trachelectomy/ Radical Trachelectomy + SLN/Lymphadenectomy (EXP)	Neoadjuvant CT + Cone Biopsy/Simple Trachelectomy/ Radical Trachelectomy + SLN/Lymphadenectomy (EXP)	Radical Hysterectomy (Type C) + SLN/Lymphadenectomy	Radical Hysterectomy (Type C) + SLN/Lymphadenectomy
**Stage IIA1** **(~≤4 cm)**	---	----	Radical Hysterectomy (Type C)	Radical Hysterectomy (Type C) + SLN/Lymphadenectomy

* Conservative surgery criteria (ConCerv): negative cone margins (if positive, re-conization or trachelectomy), squamous cell (any grade) or adenocarcinoma (grade 1 or 2), tumor size ≤2 cm, depth of invasion ≤10 mm on LEEP/cone, negative imaging for locoregional disease; **^ Conservative surgery criteria (SHAPE):** no LVSI (preferred), negative cone margins (preferred), squamous cell/adenocarcinoma adenosquamous carcinoma (any grade), tumor size ≤2 cm, depth of invasion <10 mm on cone or MRI with <50% cervical stromal invasion, negative imaging for metastatic disease. **^&^ if margins positive for carcinoma in cone**. **^#^ not meeting conservative surgery criteria**. FIGO, International Federation of Gynecology and Obstetrics; LVSI, lymphvascular space invasion; SLN, sentinel lymph node; LEEP, loop electrosurgical excision procedure; MRI, magnetic resonance imaging; EXP: experimental.

## 3. Fertility-Sparing Treatment

Forty-three percent of patients diagnosed with cervical cancer are under 45 years of age [36]. The trend of delayed childbearing combined with the frequent young age of cervical cancer onset has resulted in a growing number of patients eligible for fertility-sparing treatment in early-stage cervical cancer. In 2018, a review of the National Cancer Database showed that in the United States, trachelectomy rates increased from 1.5% to 3.2% between 2004 and 2014 (*p* < 0.001). Notably, the greatest increase was observed in patients under 30 years of age, rising from 4.6% in 2004 to 17.0% in 2014 (*p* < 0.001) [37].

Fertility-preserving surgical options involve different techniques such as conization, simple trachelectomy, vaginal and abdominal radical trachelectomy, as well as laparoscopic or robotic radical trachelectomy—all performed alongside lymph node evaluation. In selected cases, neoadjuvant chemotherapy followed by surgical resection (via conization or trachelectomy) has been proposed and is currently being investigated. Nevertheless, this strategy is not yet endorsed by clinical guidelines due to limited evidence [38]. The adoption of MIS reduced the comorbidities linked to this procedure; however, the unexpected result of the LACC trial [3] raised a very important concern regarding the oncologic outcomes of patients undergoing radical trachelectomy by the MIS approach. The IRTA trial [39], a multicenter retrospective registry study conducted across 18 centers in 12 countries, compared 4.5-year disease-free survival between open and minimally invasive radical trachelectomy in patients with early-stage cervical cancer and tumors ≤2 cm. Of the 646 patients who met the eligibility criteria and were included in the final analysis, 358 underwent open surgery, and 288 received minimally invasive surgery. The study found no statistically significant difference in 4.5-year disease-free survival between the two approaches (*p* = 0.42). Overall survival rates at 4.5 years were similarly high: 99.2% (95% CI, 97.6–99.7) for open surgery and 99.0% (95% CI, 79.0–99.8) for MIS.

The selection of candidates for fertility-sparing treatment relies on several key criteria, including the absence of nodal metastasis, tumor size (≤2 cm), histologic subtype, depth of stromal invasion, and the presence or absence of LVSI. According to the 2024 ESGO-ESHRE-ESGE guidelines [36], fertility-sparing approaches are recommended for patients with FIGO 2018 stage IA1 to IB1 cervical cancer and tumor size <2 cm, with squamous cell carcinoma or HPV-associated adenocarcinoma. Fertility preservation is not considered standard for tumors >2 cm. The National Comprehensive Cancer Network (NCCN) guidelines [13] support offering fertility-sparing procedures to select patients with stage IA2 or IB1 disease, especially when tumors are <2 cm and meet all criteria for conservative surgery without LVSI. These patients may be treated with conservative surgery (cone or simple trachelectomy) and lymph node assessment. Radical trachelectomy remains a viable fertility-preserving surgical option for patients with stage IA1–IA2 cervical cancer with LVSI, for selected patients with stage IB1 who do not meet the criteria for less radical conservative surgery, and in select cases of FIGO 2018 stage IB2 with tumors 2–4 cm in size. The optimal management of women with lesions >2 cm who wish to preserve fertility remains undefined. An upfront abdominal radical trachelectomy procedure is a possibility. However, a higher proportion (approximately 69%) of patients in this subgroup may require radical hysterectomy and/or chemoradiation, which could ultimately compromise fertility. Reported rates of fertility preservation, lymph node involvement, and the need for adjuvant treatment vary widely across studies, ranging from 10% to 45% [40,41,42,43]. Neoadjuvant chemotherapy followed by conization is emerging as a promising strategy for patients with tumors between 2 and 4 cm. A systematic review analyzing patients with cervical tumors >2 cm who received neoadjuvant chemotherapy followed by fertility-sparing surgery suggested that this treatment strategy may offer the possibility of preserving fertility while maintaining acceptable oncologic outcomes [44]. To better define the role of neoadjuvant chemotherapy in this setting, high-quality prospective data are needed. The CONTESSA trial [45] is an ongoing multicenter, prospective, single-arm study evaluating whether neoadjuvant chemotherapy can safely reduce tumor size to enable fertility-sparing surgery in women with FIGO 2018 stage IB2 (lesions measuring 2–4 cm), node-negative. The study plans to enroll 90 eligible patients, all of whom will receive platinum-based chemotherapy. After three cycles, tumor response will be assessed through clinical examination and pelvic MRI. Patients achieving complete or partial response (residual lesion <2 cm) will be considered for fertility-sparing surgery (simple trachelectomy/large cone) (Table 2) (Figure 1).

## 4. What About the Nodes?

Lymph node status is essential for accurate staging of cervical cancer, and lymphadenectomy has long been the standard approach; however, it carries risks of complications such as lower limb lymphedema and lymphoceles. Sentinel lymph node (SLN) biopsy has become an alternative for assessing lymph node status in early-stage cervical cancer, helping reduce surgical complications while maintaining diagnostic accuracy. The SLN technique focuses on identifying and analyzing the first lymph nodes that receive drainage from the tumor area—those most likely to contain metastatic cells.

The SENTICOL I trial [8] was a prospective, multicenter study evaluating the accuracy of SLN biopsy in early-stage cervical cancer using both technetium-99m and blue dye. Patients with FIGO 2009 stage IA1 with LVSI to IB1 were included. The detection rate of at least one sentinel node was 97.8% (95% CI, 93.8–99.6%). There were 23 true-positive nodes and 2 false-negative nodes, resulting in a sensitivity of 92% (23/25; 95% CI, 74-99%) and a negative predictive value of 98.2% (111/113; 05% CI, 74–99%). When SLNs were identified bilaterally, sensitivity reached 100% (Table 1). The SENTICOL II trial [9] was a prospective, randomized, multicenter study that evaluated the impact of SLN biopsy alone versus SLN biopsy combined with pelvic lymphadenectomy on morbidity and quality of life in patients with early-stage cervical cancer (FIGO 2009 stages IA2–IIA1). SLN biopsy was associated with significantly lower lymphatic morbidity (31.4%), including lower limb lymphedema and lymphoceles, compared to those who also had pelvic lymph node dissection (51.5%, *p* = 0.0046). Additionally, neurological symptoms were less frequent in the SLN-only group (7.8% vs. 20.6%, *p* = 0.01), and patients reported better overall QOL than those who underwent full pelvic lymphadenectomy (Table 1). A post hoc analysis combining data from SENTICOL I and II evaluated oncologic outcomes in patients with early-stage cervical cancer and negative lymph nodes who underwent either SLN biopsy alone or pelvic lymphadenectomy. The findings demonstrated no statistically significant differences in disease-free survival (85.1% vs. 80.4%, *p* = 0.24) or disease-specific survival (90.8% vs. 97.2%, *p* = 0.22) between the two groups [46]. The ongoing SENTICOL III trial [47], an international, randomized, multicenter, single-blind study, is designed to compare SLN biopsy alone versus SLN biopsy followed by pelvic lymphadenectomy in terms of disease-free survival and health-related QOL in early-stage cervical cancer (FIGO 2009 stage IA1 with LVSI to IIA1). The study will randomize 900 patients to test the hypothesis that disease-free survival is not inferior and health-related QOL is superior after SLN biopsy alone compared to SLN plus pelvic lymphadenectomy.

The SENTIX/ENGOT-Cx2 trial [10] was a prospective, single-arm, multicenter cohort study that evaluated whether SLN biopsy alone, when bilateral mapping was achieved, is non-inferior to historical controls of systematic pelvic lymphadenectomy in terms of recurrence rate in early-stage cervical cancer (FIGO 2009 IA1 with LVSI positive to IB1). The 24-month recurrence rate was 6%, confirming non-inferiority to the 7% benchmark after pelvic lymphadenectomy. Additionally, a bilateral SLN detection rate of 92% was achieved, with lymph node metastases identified in over 56% of cases using frozen section analysis and in more than 90% following ultrastaging (Table 1).

The NCCN guidelines [13] recommend performing SLN assessment, which should be submitted for ultrastaging if the initial hematoxylin and eosin staining is negative. Any suspicious lymph nodes should be excised regardless of mapping results. Additionally, if mapping fails in one hemipelvis, a side-specific pelvic lymphadenectomy should be performed. Based on the 2023 ESGO guidelines [12], SLN biopsy without PLND may be considered for patients with stage IA1 with LVSI or IA2 without LVSI, and is recommended for IA2 with LVSI. For IB1 and above, SLN plus PLND is advised.

## 5. Conclusions

Despite ongoing global emphasis on prevention and screening, surgical strategies for early-stage cervical cancer have undergone meaningful transformation in recent years. Robust prospective evidence has redefined the role of radical hysterectomy, with open surgery remaining the gold standard, following data from the LACC trial. At the same time, less radical procedures—such as simple hysterectomy or conization combined with nodal assessment—have been validated as safe and effective alternatives for patients with low-risk disease, reducing surgical morbidity and preserving function. This evolution in surgical strategy reflects a broader movement toward risk-adapted, patient-centered care. In carefully selected individuals, fertility-sparing techniques and SLN mapping enable effective treatment with minimal impact on reproductive potential and QOL. These developments are especially relevant for young patients, in whom long-term survivorship and functional outcomes are critical. As the field moves toward increasingly individualized treatment, high-level evidence must continue to guide surgical decision-making. Ongoing trials and long-term follow-up will further refine the criteria for conservative management and help define the optimal surgical approach for each clinical scenario in early-stage cervical cancer.

## Abbreviations

The following abbreviations are used in this manuscript:

FIGO	International Federation of Gynecology and Obstetrics
MIS	Minimally Invasive Surgery
SLN	Sentinel Lymph Node
CTCAE	Common Terminology Criteria for Adverse Events
QOL	Quality of life
NCDB	National Cancer Database
SEER	Surveillance, Epidemiology, and End Results
LEEP	Loop Electrosurgical Excision Procedure
NCCN	National Comprehensive Cancer Network
LVSI	Lymphovascular Space Invasion
MRI	Magnetic Resonance Imaging
PLND	Pelvic Lymph Node Dissection
